# Pulsatile dry cupping in chronic low back pain – a randomized three-armed controlled clinical trial

**DOI:** 10.1186/s12906-018-2187-8

**Published:** 2018-04-02

**Authors:** M. Teut, A. Ullmann, M. Ortiz, G. Rotter, S. Binting, M. Cree, F. Lotz, S. Roll, B. Brinkhaus

**Affiliations:** Institute for Social Medicine, Epidemiology and Health Economics, Charité – Universitätsmedizin Berlin, corporate member of Freie Universität Berlin, Humboldt-Universität zu Berlin, and Berlin Institute of Health, Luisenstr. 57, 10117 Berlin, Germany

**Keywords:** Cupping, Pulsatile cupping, Minimal cupping, Low back pain, Chronic low back pain, RCT

## Abstract

**Background:**

We aimed to investigate the effectiveness of two different forms of dry pulsatile cupping in patients with chronic low back pain (cLBP) compared to medication on demand only in a three-armed randomized trial.

**Methods:**

110 cLBP patients were randomized to regular pulsatile cupping with 8 treatments plus paracetamol on demand (*n* = 37), minimal cupping with 8 treatments plus paracetamol on demand (*n* = 36) or the control group with paracetamol on demand only (n = 37). Primary outcome was the pain intensity on a visual analogue scale (VAS, 0–100 mm) after 4 weeks, secondary outcome parameter included VAS pain intensity after 12 weeks, back function as measured with the ‘Funktionsfragebogen Hannover Rücken’ (FFbH-R) and health related quality of life questionnaire Short form 36 (SF-36) after 4 and 12 weeks.

**Results:**

The mean baseline-adjusted VAS after 4 weeks was 34.9 mm (95% CI: 28.7; 41.2) for pulsatile cupping, 40.4 (34.2; 46.7) for minimal cupping and 56.1 (49.8; 62.4) for control group, resulting in statistically significant differences between pulsatile cupping vs. control (21.2 (12.2; 30.1); *p* < 0.001) and minimal cupping vs. control (15.7 (6.9; 24.4); *p* = 0.001). After 12 weeks, mean adjusted VAS difference between pulsatile cupping vs. control was 15.1 ((3.1; 27.1); *p* = 0.014), and between minimal cupping vs. control 11.5 ((− 0.44; 23.4); *p* = 0.059). Differences of VAS between pulsatile cupping and minimal cupping showed no significant differences after 4 or 12 weeks. Pulsatile cupping was also better (− 5.8 (− 11.5;-0.1); *p* = 0.045) compared to control for back function after 4 weeks, but not after 12 weeks (− 5.4 (− 11.7;0.8); *p* = 0.088), pulsatile cupping also showed better improvements on SF-36 physical component scale compared to control at 4 and 12 weeks (− 5.6 (− 9.3;-2.0); *p* = 0.003; − 6.1 (− 9.9;-2.4); *p* = 0.002). For back function and quality of life minimal cupping group was not statistically different to control after 4 and 12 weeks. Paracetamol intake did not differ between the groups (cupping vs. control (7.3 (− 0.4;15.0); *p* = 0.063); minimal cupping vs. control (6.3 (− 2.0;14.5); *p* = 0.133).

**Conclusions:**

Both forms of cupping were effective in cLBP without showing significant differences in direct comparison after four weeks, only pulsatile cupping showed effects compared to control after 12 weeks.

**Trial registration:**

The study was registered at ClinicalTrials.gov (identifier: NCT02090686).

## Background

Back pain is a very common complaint in Germany; the 12-month prevalence was reported to be 66% in women and 58% in men [1], the 1-day prevalence between 32 and 49% [2], and the prevalence over lifetime between 74 and 85% [3]. Between 5 and 10% of all patients with low back pain will develop chronic low back pain (cLBP), accompanied by high treatment costs, sick leave, and individual suffering. Low back pain is one of the main reasons that people seek health care services [4]. The prevalence increases from the third decade of life until 60 years of age and it is more prevalent in women [4]. The Global Burden of Disease 2010 study showed that globally low back pain causes more disability than any other condition, the global point prevalence was 9.4% (95% CI 9.0 to 9.8) [5].

Pharmacological treatments alone do often not lead to sufficient clinical responses, and the use of non-steroidal anti-inflammatory drugs (NSAIDs, in particular) may lead to negative side effects such as gastrointestinal or renal complications. The German National Disease Management Guideline ‘Low Back Pain’ recommends patient education, physical activity, relaxation therapy, behavioural therapy, and occupational therapy as interventions [4]. However, not all cLBP patients can be treated sufficiently and often continue to have clinically relevant symptoms. Therefore, cLPB patients often use complementary and integrative medicine (CIM) therapies like acupuncture, manual therapy, or cupping, but the effectiveness of many CIM treatments is unclear.

One of the oldest traditional therapies worldwide, and especially in the Asian, Middle-East and European medical traditions, cupping is very often used to treat musculoskeletal diseases [6, 7]. Cupping is based on a sucking traction of the skin: a cupping glass is applied to a predefined skin area and a negative pressure (compared to atmospheric pressure) is generated mechanically (pumping) or thermally (cooling heated air) withdrawing the trapped air from under the cup [7, 8]. This results in reddening and warming of the affected skin area due to increased perfusion. In “dry cupping”, a negative pressure is applied, whereas in “wet cupping” the skin under the cup is pricked with a needle and the cupping is accompanied by bleeding. A modern technology is pulsatile cupping, in which a mechanical device generates a pulsatile negative pressure with a pump [8].

Huang et al. recently investigated the effectiveness of cupping in treating low back pain in a systematic review [9]. They identified only one randomized controlled trial (RCT) (10), six non-RCTs, 20 case reports, and two other studies, concluding that cupping “is promising for pain control and improvement of quality of life, and minimises the potential risks of pharmaceutical treatments but that further studies are needed to determine the potential role of cupping therapy in the treatment of low back pain.”

Recently Wang et al. [10] published a meta-analysis on cupping in low back pain. They were able to include six RCTs and showed that cupping therapy was superior to the control therapies regarding Visual Aanalogue Scale scores (SMD: − 0.73, [95% CI: -1.42 to − 0.04]; *P* = 0.04), and ODI scores (SMD: − 3.64, [95% CI: -5.85 to − 1.42]; *P* = 0.001), but a high level heterogeneity and risk of bias limited the validity of the findings. Cupping for low back pain is also discussed in other up to date reviews [11–13].

To date, no clinical studies have been published about the effectiveness of dry cupping and especially pulsatile cupping in cLBP. The aim of our study was to investigate the effectiveness of dry pulsatile cupping in reducing pain and improving back function and quality of life in patients with nonspecific cLBP. We were additionally interested in the relationship between the strength of the applied pressure and outcomes, and whether a dose-response-relationship could be observed. We therefore designed a three-armed, randomized controlled trial comparing i) strong negative pressure pulsatile cupping plus paracetamol on demand (pulsatile cupping) vs. paracetamol on demand only (control, no cupping), and ii) weak negative pressure cupping plus paracetamol on demand (minimal cupping) vs. paracetamol on demand only (control, no cupping).

## Methods

### Design

This study was designed as a three-armed, parallel, participant blinded monocenter randomized controlled clinical trial. All study participants gave written informed consent before inclusion. The study was performed at the Charité Universitätsmedizin in Berlin, Germany, between March 2014 and February 2015. Patients who fulfilled the pre-screening criteria were invited to meet the study physician for further information, at which time informed consent was obtained, inclusion and exclusion criteria were checked, and enrolled patients received a baseline assessment. The allocation to the three treatment groups followed a 1:1:1 block randomization process with a variable block length and was performed by a study nurse not being included in the recruitment by telephone. The randomization sequence was generated by SAS 9.2 Software (SAS Institute Inc. Cary. NC, USA). Allocation to treatment was concealed.

### Patients

Participants were recruited through newspaper advertisements, the website of the outpatient department for integrative medicine, and the central email newsletter of the department. Interested patients were informed about the study, and an experienced study nurse pre-screened them on the phone for inclusion.

Inclusion criteria were patients of both gender of 18–65 years with the clinical diagnosis of nonspecific cLBP, defined as pain duration of at least 3 months and an absence of specific pathological neurological symptoms. Further inclusion criteria were self-assessed subjective pain intensity ≥40 mm on the Visual Analogue Scale (0–100 mm; VAS) for the previous week, pharmacological treatment only with NSAIDs or no treatment in the last 4 weeks, and a signed informed consent. Exclusion criteria were the use of anticoagulants (e.g. Phenprocoumon, Heparin, Apixaban), a known coagulopathy, cupping treatments within the last 6 weeks, other complementary medicine therapies in the last 12 weeks (e.g. acupuncture, osteopathy), physical therapy in the last 12 weeks (including e.g. massage, chirotherapy), participation in another study in the last 3 months, allergy to or intolerance of paracetamol, pathological neurological symptoms such as muscular paralysis or paresthesia due to spinal disc herniation or other causes, known renal and / or hepatic diseases, intake of central nervous system-acting analgesics in the last 6 weeks (e.g. opioids), application for early retirement due to low back pain, and other severe disease states that disallow participation.

### Study interventions

The study protocol was developed by an expert panel experienced in cupping patients with LBP.

**Fig. 1:**
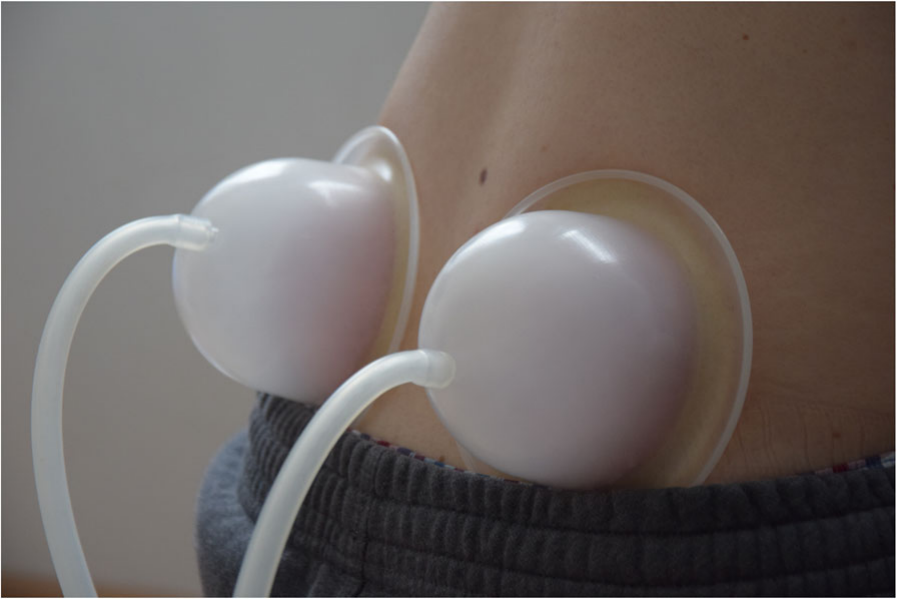
Application of the silicone cups at the low back area

Participants randomized to the pulsatile cupping group received 8 cupping sessions (each 8 min) in 4 weeks with a HeVaTech PST 30 pulsatile cupping device and a negative pressure between − 150 to − 350 mbar) and suction intervals of 2 s (see Fig. 1). In addition, paracetamol (maximum dosage 4 × 500 mg/day) on demand as rescue medication was allowed. Figure 1 shows both silicone cups being applied to the low back area.

Participants randomized to the minimal cupping group received 8 cupping sessions (each 8 min) in 4 weeks with a HeVaTech PST 30 pulsatile cupping device, also with two silicone cups and a weaker negative pressure around - 70 mbar and suction intervals of 2 s. In addition, paracetamol (maximum dosage 4 × 500 mg/day) on demand as rescue medication was allowed.

Participants randomized to the control group received no cupping intervention in the study period of 12 weeks, but were allowed to treat their back pain complaints with paracetamol (maximum dosage 4 × 500 mg/day) on demand. All patients in the control group were offered a cost-free cupping intervention after completing the trial after 12 weeks.

Specially trained medical doctors, nurses, and/or medical students applied the cupping treatments. Patients of both cupping groups were blinded to their study intervention.

### Outcome parameters

Patients completed standardized questionnaires measuring outcomes at baseline, and after 4 and 12 weeks. The primary outcome parameter was the mean of the subjective pain intensity during the week prior to treatment and again after 4 weeks, using the Visual Analogue Scale (VAS, 0–100 mm; 0 = no pain, 100 mm = maximum intensity) [14]. Secondary parameters included the last week’s pain intensity on the VAS after 12 weeks, the back function measured with the ‘Funktionsfragebogen Hannover Rücken’ (FFbH-R) [15] at 4 and 12 weeks, the health-related quality of life measured with the SF-36 questionnaire [16] at 4 and 12 weeks, the perceived effect measured with a 5-point Likert scale after 4 and 12 weeks, the intake of paracetamol within the 4 weeks intervention or waiting period (diary) and adverse events across the whole study period of 12 weeks. We also assessed patient perception about their group allocation.

### Statistical analysis

The sample size calculation was performed for the primary comparison between the cupping and the control group. An adjusted difference of 15 mm on the VAS after 4 weeks with a common standard deviation of 20 mm, given a significance level of α = 0.05, was assumed for a two-sided t-test. Based on these assumptions and a power of 85%, 33 patients per group were needed. To compensate for drop-outs, a total of 36 patients per group were included and randomized. Sample size calculation was done with nQuery Advisor 6.02.

The statistical analysis was performed using the software package SAS release 9.3 / 9.4 (SAS Institute Inc., Cary, NC, USA) and IBM SPSS Statistics Version 23. The analysis of the primary outcome was calculated using an analysis of covariance (ANCOVA) with the fixed factor treatment group adjusted for baseline value of VAS pain intensity (covariate). To adjust for three group comparisons, a hierarchical testing procedure with three steps was performed, ensuring an overall significance level of α = 0.05 (two-sided). The first step was the comparison between the cupping and the control group. In case of a significant difference, the next step was performed confirmatively; otherwise, all following steps were explorative. The second step was the comparison between the minimal cupping and the control group. Again, in the case of a significant difference the next step was confirmative, otherwise, the following step was considered explorative. The third step was the comparison between the cupping and the minimal cupping groups.

All following analyses were explorative. The analyses of the secondary endpoints and the secondary analyses of the primary endpoint were performed with a similar model, depending on the distribution and the scale of the variables, but without the hierarchical procedure.

Results were reported as adjusted means with 95% confidence intervals and the *p* value for the group comparison. All tests and confidence intervals were two-sided. All data were analyzed based on the intention-to-treat-principle (ITT) using the full analysis set (FAS) with all available data without imputing for missing values.

Adverse events are presented descriptively by frequency for each treatment group.

## Results

**Fig. 2:**
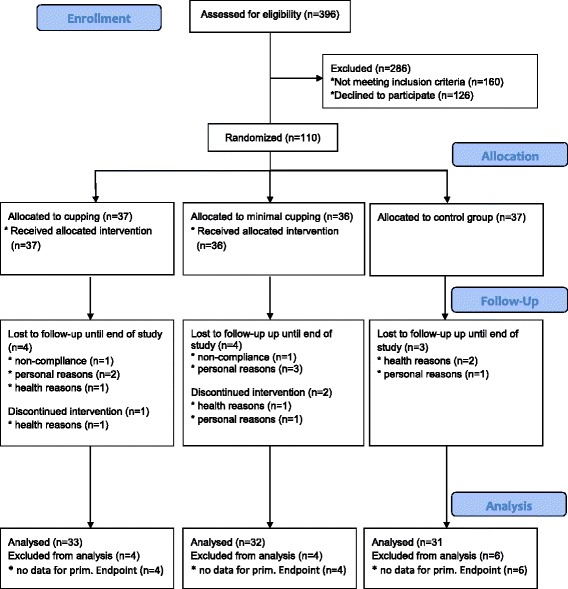
Participant flow diagram

Patients were recruited between March and September 2014. Study interventions and follow-up assessments were completed by February 2015. Figure 2 shows the recruitment and allocation process.

In total, 396 patients were screened for eligibility; 286 were excluded for not meeting the inclusion criteria (*n* = 160) or refusing to participate after being informed about the trial (*n* = 126). Altogether 110 patients were randomized and allocated to pulsatile cupping (*n* = 37), minimal cupping (*n* = 36), or control (n = 37). After 12 weeks, 11 patients (pulsatile cupping *n* = 4, minimal cupping n = 4, control n = 3) had dropped out of the trial. The main reasons for withdrawal from the study were identified as “personal” (*n* = 6). Other reasons for drop-outs were health problems (n = 3) and non-compliance (*n* = 2). Three patients discontinued the intervention because of health problems (cupping n = 1, minimal cupping n = 1) or for personal reasons (minimal cupping n = 1). The VAS data for 6 patients in the control group after 4 weeks were missing.

91.4% of the cupping group and 88.2% of the minimal cupping group had 8 cupping sessions as asked by the study protocol.

The mean age of patients was 49 years (pulsatile cupping group: 49 years, minimal cupping group: 48, control group: 51) at baseline (see Table 1); the mean Body Mass Indexes scored between 25 and 26 kg/m^2^ (Table 1). There were more males in the pulsatile cupping group (43.2%) compared to minimal cupping (36.1%) and control (32.4%). The pain intensity measure by the VAS was higher in the minimal cupping 60.3 ± 12.3 and in the control group 59.9 ± 12.8 compared with the pulsatile cupping group (53.2 ± 7.4).

**Table 1:** Baseline characteristics of patients

	Cupping High pulsatile vacuum *n* = 37 Mean ± SD / n (%)	Minimal Cupping Low pulsatile vacuum *n* = 36 Mean ± SD / n (%)	Control n = 37 Mean ± SD / n (%)
Age [years]	49.0 ± 13.7	47.5 ± 13.8	50.7 ± 10.7
Gender (Male)	16 (43.2)	13 (36.1)	12 (32.4)
BMI	26.3 ± 4.3	25.0 ± 4.1	25.3 ± 4.7
Exercise (yes)	28 (75.7)	29 (80.6)	29 (78.4)
Duration of low back pain [years]	13.1 ± 9.3	15.8 ± 12.9	13.2 ± 11.2
Current drug intake because of low back pain	11 (29.7)	14 (38.9)	14 (37.8)
Current consultations because of low back pain	35 (94.6)	36 (100)	36 (97.3)
VAS pain intensity [mm]^a^	53.2 ± 7.4	60.3 ± 12.3	59.9 ± 12.8
FFbH-R^b^	75.9 ± 16.3	72.2 ± 12.9	70.2 ± 18.7
SF-36			
Physical Component Summary^b^	39.1 ± 8.4	38.2 ± 6.6	38.6 ± 8.5
Mental Component Summary^b^	50.9 ± 10.4	50.2 ± 9.1	50.1 ± 10.0

^a^Lower values indicate better status, ^b^higher values indicate better status

**Fig. 3:**
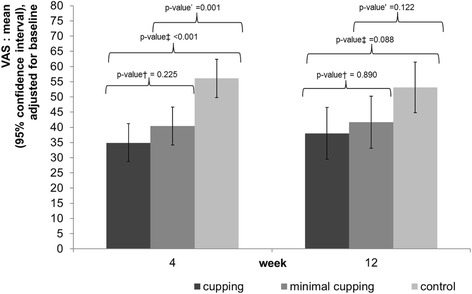
Pain intensity measured by Visual Analogue Scale at 4 and 12 weeks. Legend Fig. 3: VAS, 0–100 mm; 0 = no pain, 100 mm = maximum intensity †low vacuum vs high vacuum, ‡ control vs high vacuum, ‘control vs low vacuum

The mean adjusted VAS pain intensity after 4 weeks was 34.9 mm (95% CI: 28.7; 41.2) for the pulsatile cupping group, 40.4 (34.2; 46.7) for minimal cupping, and 56.1 (49.8; 62.4) for control (see Table 2 and Fig. 3), resulting in statistically significant differences between pulsatile cupping vs. control (21.2 (12.2;30.1), *p* < 0.001) and minimal cupping vs. control (15.7 (6.9;24.4), *p* = 0.001) (Table 2, Fig. 3).

**Table 2:** Primary and secondary outcomes at week 4: group means and mean group differences with 95% confidence interval (CI), adjusted for respective baseline value

	Cupping (*n* = 33)	Minimal Cupping (*n* = 32)	Control (*n* = 31)	Control vs. Cupping		Control vs. Minimal Cupping		Minimal Cupping vs. Cupping	
	Mean (95% CI)	Mean (95% CI)	Mean (95% CI)	Mean difference (95% CI)	*p*-value	Mean difference (95% CI)	*p*-value	Mean difference (95% CI)	*p*-value
VAS pain intensity^a^(primary outcome)	34.9 (28.7;41.2)	40.4 (34.2; 46.7)	56.1 (49.8;62.4)	21.2 (12.2;30.1)	< 0.001	15.7 (6.9;24.4)	0.001	5.5 (−3.5; 14.5)	0.225
FFbH-R^b^	76.0 (72.1;80.0)	74.2 (70.2;78.2)	70.2 (66.1;74.3)	-5.8 (−11.5;-0.1)	0.045	−4.0 (−9.7;1.7)	0.171	−1.8 (−7.5;3.8)	0.517
SF-36^b^									
Physical Component Summary	43.8 (41.3;46.4)	39.5 (37.0;42.1)	38.2;(35.6;40.9)	−5.6 (−9.3;-2.0)	0.003	−1.3 (−5.0;2.4)	0.478	−4.3 (−7.9;-0.7)	0.021
Mental Component Summary	50.1 (47.0;53.2)	47.1 (44.0;50.3)	47.6 (44.4;50.8)	−2.5 (−6.9;2.0)	0.270	0.5 (−4.0;5.0)	0.820	−3.0 (−7.4;1.4)	0.179
SF-36 subscales^b^									
General health	63.2 (58.8;67.6)	55.2 (50.6;59.8)	56.8 (52.2;61.4)	−6.4 (−12.7;0.02)	0.051	1.7 (−4.9;8.2)	0.617	−8.0 (− 14.4;-1.6)	0.015
Mental health	70.8 (66.3;75.2)	66.2 (61.7;70.8)	65.8 (61.2;70.4)	−5.0 (− 11.4;1.5)	0.128	− 0.4 (−6.9;6.0)	0.897	−4.5 (−10.9;1.8)	0.160
Bodily pain	56.1 (50.8;61.4)	48.9 (43.5;54.3)	42.3 (36.9;47.8)	−13.7 (−21.4;-6.1)	0.001	−6.5 (− 14.2;1.1)	0.093	−7.2 (− 14.7;0.4)	0.063
Physical functioning	74.7 (68.4;81.0)	69.3 (62.9;75.7)	68.8 (62.3;75.2)	−6.0 (−15.0;3.1)	0.192	−0.6 (−9.7;8.6)	0.905	−5.4 (− 14.4;3.6)	0.234
Role: Emotional	79.3 (67.6;91.0)	69.2 (57.3;81.1)	70.1 (57.8;82.4)	−9.2 (−26.2;7.8)	0.284	0.9 (−16.2;18.0)	0.918	−10.1 (−26.8;6.6)	0.233
Role: Physical	68.0 (56.4;79.6)	48.6 (36.8;60.3)	45.3 (33.3;57.2)	−22.7 (−39.4;-6.1)	0.008	−3.3 (− 20.1;13.5)	0.699	−19.4 (− 35.9;-3.0)	0.021
Social functioning	79.8 (73.1;86.5)	69.1 (62.3;76.0)	72.4 (65.5;79.3)	−7.4 (−17.0;2.2)	0.131	3.3 (−6.5;13.0)	0.510	−10.6 (−20.2;-1.0)	0.03
Vitality	53.8 (49.4;58.2)	46.5 (42.1;50.9)	44.4 (39.9;48.9)	−9.3 (−15.6;-3.0)	0.004	−2.0 (−8.4;4.3)	0.526	−7.3 (−13.5;-1.1)	0.022
Pain rescue medication (count of pills, Paracetamol)	4.6 (−1.0;12.0)	5.6 (−0.7;12.0)	11.9 (6.7;17.1)	7.3 (−0.4;15.0)	0.063	6.3 (−2.0;14.5)	0.133	1.0 (−7.5;9.5)	0.814

^a^Lower values indicate better status, ^b^higher values indicate better status

After 12 weeks, mean adjusted VAS pain intensity was lower for pulsatile cupping vs. control (15.1 (3.1;27.1), *p* = 0.014), but not for minimal cupping vs. control (11.5 (− 0.44;23.4), *p* = 0.059). The group differences of pain intensity between minimal cupping and pulsatile cupping showed no significant differences after 4 and 12 weeks (VAS adjusted mean: 5.5 (− 3.5; 14.5); *p* = 0.225) and 12 weeks (3.7 (− 8.6;15.9); *p* = 0.554). (Table 3, Fig. 3).

**Table 3:** Secondary outcomes at week 12: group means and mean group differences with 95% confidence interval (CI), adjusted for respective baseline value

	Cupping (n = 33)	Minimal Cupping (n = 32)	Control (n = 31)	Control vs. Cupping		Control vs. Minimal Cupping		Minimal Cupping vs. Cupping	
	Mean (95% CI)	Mean (95% CI)	Mean (95% CI)	Mean difference (95% CI)	*p*-value	Mean difference (95% CI)	*p*-value	Mean (95% CI)	*p*-value
VAS pain intensity^a^	38.0 (29.5;46.5)	41.7 (33.1;50.2)	53.1 (44.8;61.4)	15.1 (3.1;27.1)	0.014	11.5 (− 0.44;23.4)	0.059	3.7 (−8.6;15.9)	0.554
FFbH-R^b^	76.0 (71.6;80.4)	75.6 (71.1;80.1)	70.6 (66.2;75.0)	−5.4 (−11.7;0.8)	0.088	−5.0 (− 11.3;1.4)	0.122	−0.4 (−8.8;5.9)	0.890
SF-36^b^									
Physical Component Summary	44.9 (42.3;47.6)	41.1 (38.3;43.8)	38.8 (36.1;41.1)	−6.1 (−9.9;-2.4)	0.002	−2.3 (−6.1;1.5)	0.237	−3.8 (−7.7;-0.03)	0.048
Mental Component Summary	46.7 (43.2;50.1)	45.8 (42.2;49.3)	48.4 (45.0;51.8)	1.8 (−3.1;6.6)	0.477	2.6 (−2.3;7.6)	0.291	−0.9 (−5.8;4.0)	0.719
SF-36 subscales^b^									
General health	61.0 (56.5;65.5)	56.2 (51.5;60.9)	51.6 (47.1;56.1)	−9.4 (−15.8;-3.0)	0.004	−4.6 (−11.2;2.0)	0.167	−7.8 (− 11.3;1.8)	0.152
Mental health	68.6 (63.6;73.6)	64.8 (59.7;70.0)	67.4 (62.4;72.4)	−1.2 (−8.3;5.8)	0.732	2.4 (−4.6;9.7)	0.486	−3.7 (−10.9;3.4)	0.302
Bodily pain	58.1 (51.7;64.6)	48.9 (42.2;55.5)	46.0 (39.5;52.3)	−12.2 (−21.3;-3.1)	0.009	−2.9 (− 12.1;6.4)	0.539	−9.3 (− 18.5;-0.03)	0.049
Physical functioning	75.2 (68.3;82.1)	74.3 (67.2;81.4)	67.4 (60.5;74.3)	−7.8 (−17.5;1.9)	0.114	−6.9 (−16.9;3.0)	0.167	−0.9 (− 10.8;9.1)	0.863
Role: Emotional	69.0 (55.9;82.1)	68.5 (54.9;82.1)	73.0 (59.9;86.2)	4.1 (−14.5;22.6)	0.665	4.5 (−14.4;23.4)	0.634	−0.5 (−19.4;18.4)	0.959
Role: Physical	71.0 (58.8;83.3)	49.8 (37.2;62.5)	55.0 (42.7;67.2)	−16.1 (−33.4;1.3)	0.069	5.1 (−12.5;22.7)	0.563	−21.2 (− 38.8;-3.6)	0.019
Social functioning	75.0 (67.4;82.5)	70.4 (62.5;78.2)	74.7 (67.1;82.3)	−0.3 (−11.0;10.5)	0.960	4.3 (−6.7;15.4)	0.435	−4.6 (−15.5;6.3)	0.403
Vitality	50.9 (45.5;56.4)	47.8 (42.2;53.4)	46.8 (41.4;52.2)	−4.1 (−11.8;3.5)	0.286	−1.0 (−8.8;6.8)	0.799	−3.2 (− 10.9;4.6)	0.424

^a^Lower values indicate better status, ^b^higher values indicate better status

The FFbH-R back function of the pulsatile cupping group showed better effects than control after 4 weeks, but not after 12 weeks; the minimal cupping group was not statistically different to control after 4 and 12 weeks. The pulsatile cupping group also showed improvements on the SF-36 Physical Component Summary compared to control at 4 and 12 weeks, but not in the comparisons between minimal cupping vs. control. Also, several SF-36 sub-scores showed improvements after 4 and 12 weeks in favour of pulsatile cupping, but not of minimal cupping compared to control (e.g., bodily pain, SF-36 physical role and vitality, SF-36 General Health Perception). No differences between groups were observed for the SF-36 Mental Component Summary after 4 and 12 weeks.

Paracetamol intake did not differ between the groups (cupping vs. control (7.3 (− 0.4;15.0); *p* = 0.063); minimal cupping vs. control (6.3 (− 2.0;14.5); *p* = 0.133) (Table 2).

After four weeks, 42% of the pulsatile cupping group and 44% of the minimal cupping group rated the cupping therapy as effective on the Likert Scale, whereas around 30% in both groups said that the cupping therapy was less effective. That assessment of treatment effect remained nearly the same after 12 weeks. Most of the participants in the minimal cupping group (84%) were able to identify their group allocation after 4 weeks, whereas in the cupping cupping group 55% identified their group allocation.

No serious adverse events were observed during the whole study period. Moderate adverse events were observed in two patients in the pulsatile cupping group who reported an aggravation of their low back pain after the cupping sessions for a few hours. One of those patients dropped out of the therapy but not the study. Other reported side effects in a 24-h time interval after cupping included light muscular backache in six patients in the pulsatile cupping group, and in two patients in the minimal cupping group.

## Discussion

Both forms of cupping were effective in reducing cLBP after 4 weeks compared to the control group that only took pain medication on demand. However, there were no significant differences between pulsatile cupping and minimal cupping after 4 weeks. After 12 weeks only the pulsatile cupping group showed effects compared to control in most of the outcome parameters. However, power and sample size calculations were based on the differences to the control group, not on changes between interventions. We also observed improvements in quality of life on the SF 36 Physical Component Summary in the pulsatile cupping group after 4 and 12 weeks. Those improvements can be mainly found in the bodily pain, physical role, general health perception, and vitality subscale aspects. After 12 weeks, differences were reported only for the aspects of general health perception and bodily pain.

To our knowledge, this was the first study that compared pulsatile cupping plus medication on demand and minimal cupping plus medication on demand with the control condition medication on demand only.

Strengths of the study were the inclusion of a control group to assess the overall effect of both forms of cupping and the strict randomization and allocation process.

A major limitation of our study, as in all other cupping studies, is the lack of blinding between cupping and control interventions, which may have had influence on the study results e.g. in better results of the verum treatment and worser results in the minimal cupping group. Patients sense the application of cups and also the generation of negative pressure involved. They do also have some sense of how strong the cupping pressure applied is. A sham cupping device would be highly useful to experimentally distinguish specific from nonspecific effects of cupping. Up to 2016, no trial on sham cupping was published [17]. Lauche et al. [18] introduced 2016 a sham cupping device consisting of conventional cupping glasses being fixed on the skin with elastic tape. The cups were prepared with small holes, through which the negative pressure was released during seconds after the negative pressure was applied. This is a clever solution, but also this sham device relies on short period of cupping, as an application of negative pressure is involved. In our own preparation for this trial, we experimented with different systems and developed a special form of minimal cupping using a pulsatile cupping device and applied the weakest negative pressure being able to fix the silicone cups on the skin. However, our procedure yielded no sufficient blinding, especially in the minimal cupping group. Any minimal cupping procedure restricting cupping time or reducing the pressure involved may produce some specific effects and would therefore better be called “minimal cupping” instead of sham.

In our trial, the no cupping control group may be seen as a limitation, as no active intervention was undertaken. In our opinion, this limitation is debatable and ethically justifiable because all patients were allowed up to 2 g of paracetamol per day as pain medication on demand. In the waiting control there may have been an element of frustration of the participants about the allocation to no active intervention, which could have had a negative influence on the outcome assessments. We tried to minimize this possible element of bias by offering all control group patients a complete set of eight cost free cupping therapies after 12 weeks (after the end of the trial). However, theoretically, this potential bias could also explain differences between active therapy and control.

We based our sample size calculation on a small difference of 15 mm on the VAS because we assumed that this would make a clinically important change between both cupping groups compared to the control group. Based on our assumption, this would mean that there were clinically relevant changes for pulsatile cupping and minimal cupping after 4 weeks and for pulsatile cupping after 12 weeks. Other authors have described the minimal clinical important change of VAS in chronic low back pain higher around 20 mm [19, 20]. In this case, only pulsatile cupping would show a relevant improvement vs. control after 4 weeks, but not minimal cupping.

Only few studies have been published investigating the effects of cupping on low back pain: One RCT by Kim et al. [21] investigated the effectiveness of six wet cupping sessions compared to a no therapy (waiting list) control group in 32 Korean participants. Significant differences regarding pain intensity on the McGill Pain Questionnaire for pain intensity and reduction of acetaminophen intake were described after 2 weeks and 4 weeks, but there was no significant difference for pain intensity on a numeric rating scale and the Oswestry Disability Questionnaire.

Another RCT of Farhadi et al. (2009) [22] compared 3 sessions of wet (bloody) cupping in one week to usual care in 98 Iranian patients with nonspecific low back pain and observed significant group differences on the McGill Present Pain Index, the Owestry Pain Disability Index and on the Medication Quantification Scale after 3 months. Farhadi et al. included patients with a pain duration longer than 4 weeks, so their sample could have been different from ours. They also used a different (blood sucking) technique and used different areas of cupping.

The effects of both cupping interventions on VAS intensity of pain compared to waiting control group in our trial are very comparable to the effects we observed in a former trial of twelve sessions of acupuncture or minimal acupuncture compared to waiting control group in patients with chronic low back pain [23]. From the clinicians view dry cupping may be seen as a less invasive form of reflex therapy compared to acupuncture with needles. A mix of potential effectors of cupping was suggested by Musial et al. [24] who generally proposed three potential mechanisms of action for reflex therapies such as cupping: (1) pain reduction could be caused by deforming or even injuring the skin which may stimulate Aβ fibres in painful skin regions, (2) manipulations may stimulate inhibitory receptive fields of the multi-receptive dorsal horn neurons, and (3) the setting may have a relaxing and socially comforting effect. Emerich et al. who did research on the local reactions in the cupped areas described a strong anaerob metabolism with high lactate concentrations in the regions being cupped [25]. Cupping induced a lasting anaerobe metabolism in the subcutaneous tissue and did increase immediate pressure pain thresholds in some areas.

Although we find evidence that cupping is effective in cLBP, our data does not allow conclusions about specific mechanisms or effectors of cupping. Also in the Lauche et al. trial, no specific effect of the verum cupping could be detected compared to the described sham cupping device in patients with chronic neck pain. The mechanism of cupping remains unclear, effects could as well be caused by unspecific effects and expectation, especially in a waiting group design trial. However, as dry cupping is a non-pharmacological and comparably safe therapy it may be of use in clinical care independent of mechanisms involved. Further research about mechanisms involved in cupping, specific effects but also real life effects in clinical care routine conditions are needed to further understand its mode of action and its usefulness.

## Conclusion

Both forms of cupping were effective in patients with chronic low back pain after 4 weeks without showing significant differences in direct comparison. In addition, only pulsatile cupping showed effects compared to a non-treatment control in reducing pain after 12 weeks, but not minimal cupping.

## Abbreviations

BMI: Body Mass Index
CIM: complementary and integrative medicine
cLBP: chronic low back pain
FAS: full analysis set
FFbH-R: Funktionsfragebogen Hannover zu Funktionsbeeinträchtigung durch Rückenschmerzen
ITT: intention-to-treat
NSAID: non-steroidal anti-inflammatory drugs
RCT: randomized controlled trial
SF-36: Short Form-36
VAS: Visual Analogue Scale
